# Memory’s double take: Dissociating two forms of recollection in visual working memory

**DOI:** 10.3758/s13421-026-01890-6

**Published:** 2026-05-28

**Authors:** Stephanie Norris, Andrew P. Yonelinas

**Affiliations:** 1 Center for Neuroscience, University of California, Davis, CA, USA; 2 Department of Psychology, University of California, Davis, CA, USA

**Keywords:** Dual-process, Recollection, Familiarity, Perceiving, Sensing, Visual working memory, Receiver operating characteristics

## Abstract

Visual working memory (VWM) supports both change detection and repetition detection, yet it remains unclear whether these operations can be functionally dissociated. We compared two VWM paradigms designed to isolate each process. The *Any-Difference* test presents an array of five colored squares, a brief retention interval, and then a test array in which either all squares retain their color or one square changes its shade; participants indicate whether any change occurred. The *Any-Sameness* test uses the same stimulus sequence but has participants report whether any square is a repeat of a previously shown item. In Experiment 1 (*N* = 24), participants provided six-point confidence ratings, and receiver operating characteristics (ROCs) were fit with a dual-process signal detection model, yielding estimates of recollecting a change, recollecting a repetition, and familiarity-driven discrimination (*d′*). The *Any-Difference* test produced steep, curvilinear ROCs with higher recollection estimates for changes than repetitions, whereas the *Any-Sameness* test produced shallower ROCs with higher recollection estimates for repetitions. Experiment 2 (*N* = 28) added subjective reports in which participants indicated whether they had actively maintained an item and “perceived” a change/repetition, or merely “sensed” it. ROC patterns replicated Experiment 1 and subjective reports confirmed that recollection estimates corresponded to trials involving active maintenance. Together, these findings show that the two tests provide complementary leverage on change- and repetition-based judgments and that the ability to recollect changes can be dissociated from recollecting repetitions. These methods offer a new approach for probing the underlying VWM processes in both healthy and clinical populations.

## Introduction

Visual working memory (VWM) temporarily stores and manipulates visual information, serving as an interface between perception, attention, and long-term memory (Awh et al., 2006; Cowan, 2008; Hitch et al., 2024; Luck & Vogel, 2013). This adaptive system lets us navigate complex, rapidly changing visual environments (Orhan et al., 2014). Two core functions of VWM are detecting when something has changed and recognizing when something has repeated. In everyday situations – such as driving – this distinction is behaviorally significant: noticing an unexpected change can trigger immediate attention and action, whereas recognizing a repetition reassures us that no intervention is needed. Despite their importance, change detection and repetition detection are often overlooked in working memory research; in fact, many studies focus solely on assessing overall working memory capacity or strength, thereby collapsing across these two abilities into aggregate measures such as proportion correct, capacity, or sensitivity (e.g., *d’*).

There is, however, a growing literature showing that working memory can draw on multiple underlying processes – most notably recollection and familiarity (Aly & Yonelinas, 2012; Awh & Vogel, 2025; Cowan et al., 2021; Lin & Oberauer, 2022; Yonelinas, 2024). These ideas stem from earlier dual-process accounts of episodic memory, such as those proposed by Larry Jacoby (Atkinson & Juola, 1974; Jacoby, 1991; Jacoby & Dallas, 1981; Mandler, 1980; Norman & O’Reilly, 2003). The core assumption underlying these models is that memory judgments can be based either on assessments of familiarity (i.e., how well the retrieval cue matches what has been stored in memory) or on recollection (i.e., the recall of qualitative information about an earlier study event). Similar processes have been proposed to support performance on working memory tests, but with one critical distinction. In working memory, recollection is generally thought to rely on active maintenance or rehearsal (see also work on “refreshing” by Johnson et al., 2002, and Raye et al., 2007). In episodic memory, by contrast, items cannot be actively maintained across long delays and numerous interfering events, so recollection instead relies on retrieval – or re-accessing – of information from episodic memory. Importantly, if recollection contributes to working memory, it should support performance in two different ways – by enabling the detection of changes and the detection of repetitions. For example, if a subject is actively maintaining that there is a red square in the upper left corner of the display, then noticing that the square has changed to green at test allows them to use recollection to identify a change. Conversely, if they notice that the square remained red, they can use recollection to identify a repetition.

In a recent working memory study, we found evidence that the probability of recollecting changes and recollecting repetitions differed systematically across different types of working memory tests (Norris & Yonelinas, 2025). In that study, each trial began with participants encoding an array of five colored squares. After a brief delay, they completed a recognition test in which they rated their memory confidence, under three different testing conditions. The study revealed distinct patterns of recollection estimates across the three tests. In a *complex-probe* test, participants judged whether an entire test array of colored squares was a repeat of the study array or contained a color change; performance in this condition depended more heavily on recollecting changes than on recollecting repetitions. By contrast, an *item recognition* test required participants to decide whether a centrally presented color had been part of the study array, and here performance relied more on recollecting repetitions than on recollecting changes. Finally, in a *single-probe* test, participants indicated whether a single-colored square presented at a specific location had been studied; performance in this condition drew on both recollecting repetitions and recollecting changes.

The observed dissociations align with the specific demands of each test. For example, in the *complex-probe* test, performance hinges on change detection: correctly identifying a change requires remembering that a specific item was altered, which alone yields a correct “different” response. By contrast, merely recalling that an item was repeated is insufficient because another item could still have changed. Conversely, in the *item recognition* test, a confident “same” response depends on recollecting that the probe was indeed repeated – this alone supports a correct “same” response on repeated trials. Detecting a “different” trial, however, requires the participant to recollect all studied items to ensure that none match the probe, a demand that is much less likely to be met. In the *single-probe* test, maintaining the color of the probed item supports both accepting an old item as old and rejecting a new item as having changed; neither decision depends on memory for the entire study array.

Overall, these results indicated that change detection and repetition detection contribute differentially to distinct types of working memory tests. Moreover, they suggested that these differences arose because the tests differed in the extent to which they required that subjects recollected changes and/or repetitions. However, there were potential limitations to that study that we aimed to address in the current set of experiments.

### The current study

The current study was designed to address two questions. First, can the roles of change detection and repetition detection be dissociated in tests designed to selectively manipulate these processes? The previous study found evidence that these two forms of memory can be dissociated across different types of working memory tests, but those tests differed in several ways beyond their reliance on repetition detection and change detection. For example, the *complex-probe* test required participants to remember the color that had occurred in a specific location, whereas the *item recognition* test required only color memory. Moreover, in the *complex-probe* test, a full array served as the retrieval cue, whereas in both the *item recognition* and *single-probe* tests, single items were used as retrieval cues. Thus, the observed dissociations may have been influenced, at least in part, by these procedural differences rather than by the differential roles of change detection and repetition detection per se.

**Fig. 1:**
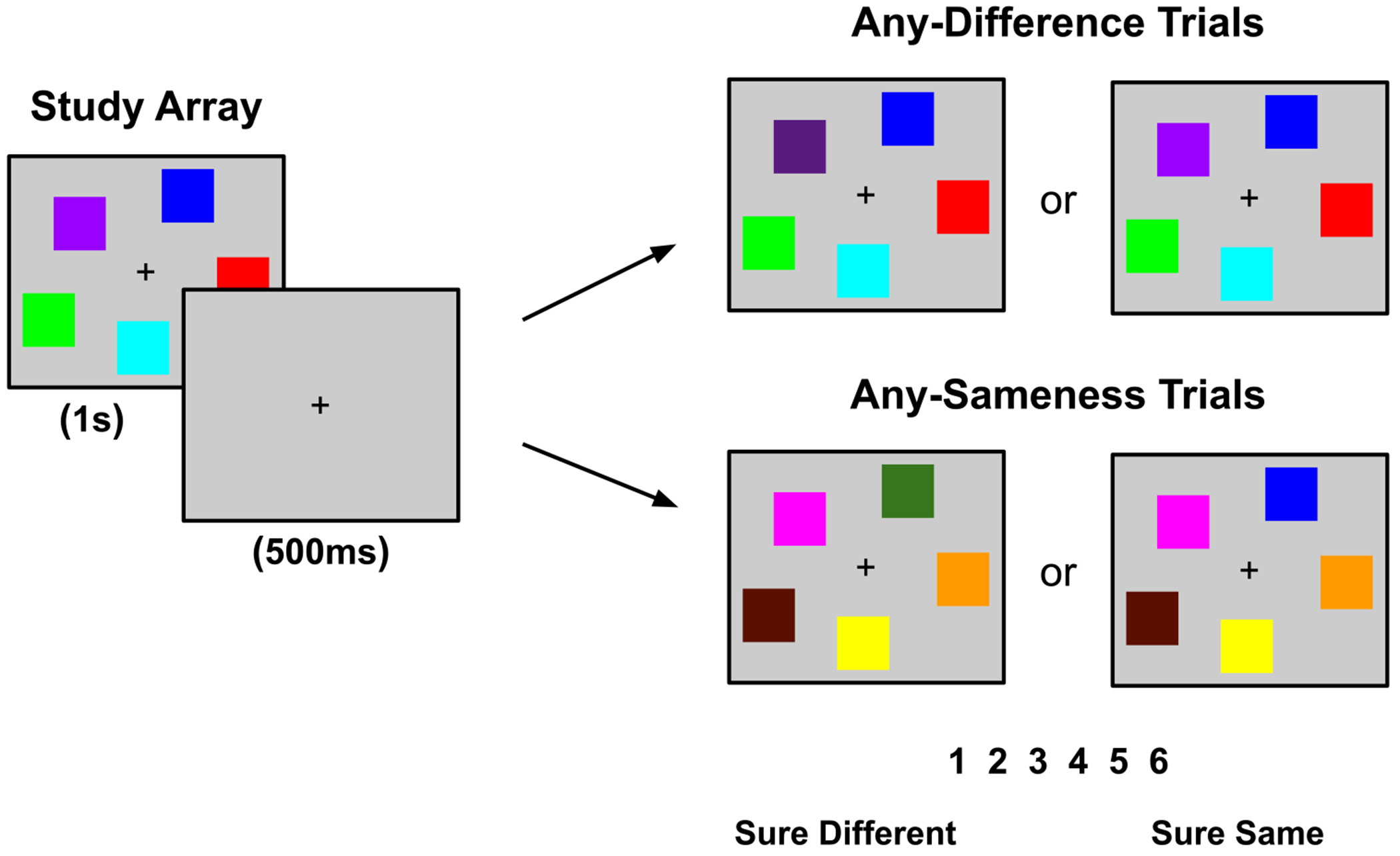
Illustration of experimental design*.* On each trial, participants studied an array of five colored squares and, after a brief delay, viewed a test array and made a same/different confidence judgment. In one block, they completed the *Any-Difference* test (upper right panel); in the other, they completed the *Any-Sameness* test (lower right panel). Example “same” and “different” trials are shown for each test type

To more directly manipulate the contributions of change and repetition detection, we examined performance in an *Any-Difference* and an *Any-Sameness* test to isolate these distinct forms of recollection (see Fig. 1). The experiments were based on an approach introduced by Taylor (1976),^1^ who presented participants with two side-by-side arrays containing four letters and asked them to judge whether any items had changed (*Any-Difference*) or whether any items had remained the same (*Any-Sameness*). Although the original study focused on multiletter matching, the manipulation has also been applied to working memory tests in which a delay is introduced between the two arrays (Hyun et al., 2009; Jiang et al., 2000).

The *Any-Difference* test parallels the *complex-probe* test described earlier. Participants studied an array of colored squares and, after a brief retention interval, viewed a second array in which either no change occurred or one item changed to a different shade of its original color. Importantly, the categorical color of the changed item was preserved, and no new colors were introduced at test; thus, the manipulation involved within-color shade changes rather than changes to a categorically different color. This subtle change was implemented because detecting a change is typically easier than detecting a repetition (Hyun et al., 2009) and served to reduce – though not fully eliminate – performance differences between the two tests. The *Any-Sameness* test followed an identical trial structure but differed in the nature of the test array. To avoid making repetition detection prohibitively difficult, no shade changes were used in this condition. On “different” trials, none of the colors in the first array reappeared in the second array. On “same” trials, four items were replaced with unseen colors, and one item repeated the exact same color as in the study array. Thus, the *Any-Sameness* test required participants to detect the presence of a repeated color, whereas the *Any-Difference* test required detection of a within-color change. Critically, both tests involved memory for color and location information and used a complex array as the retrieval cue.

The second potential concern with the earlier study was that only one method (i.e., ROC modeling of confidence responses) was used to measure the contributions of recollection and familiarity. Although the ROC model fit the observed data well and has been useful in many previous studies (Yonelinas, 2024, 2025; Yonelinas & Parks, 2007), it relies on several assumptions that can be violated (e.g.,Wixted, 2007; Yonelinas & Parks, 2007). Therefore, it is important to test whether other measurement approaches converge on the same conclusions. In Experiment 1 of the current study, we used the same ROC model to derive estimates of recollection and familiarity as in Norris and Yonelinas (2025). In Experiment 2, however, to assess the generalizability of the initial results, we included a “perceive/sense” subjective report method (Aly & Yonelinas, 2012; Ramey et al., 2022; Rensink, 2000). With this approach, participants were asked to indicate whether their working memory judgments were based on recollection (i.e., they recollected the original color and “perceived” that it had changed/repeated) or on familiarity (i.e., they just “sensed” that there was a change/repetition in the absence of recollection). Based on previous working memory studies using scenes (Aly & Yonelinas, 2012) and faces (Goodrich et al., 2019), we expected that estimates based on the ROC results would converge with those based on the subjective reports. However, whether this convergence would also occur with relatively simple color stimuli examined here is unknown, as it is conceivable that the subjective experiences of “perceiving” and “sensing” may be less accessible to awareness when dealing with lower-level visual features like colors.

### Using confidence judgments and ROC analysis to assess visual working memory

**Fig. 2:**
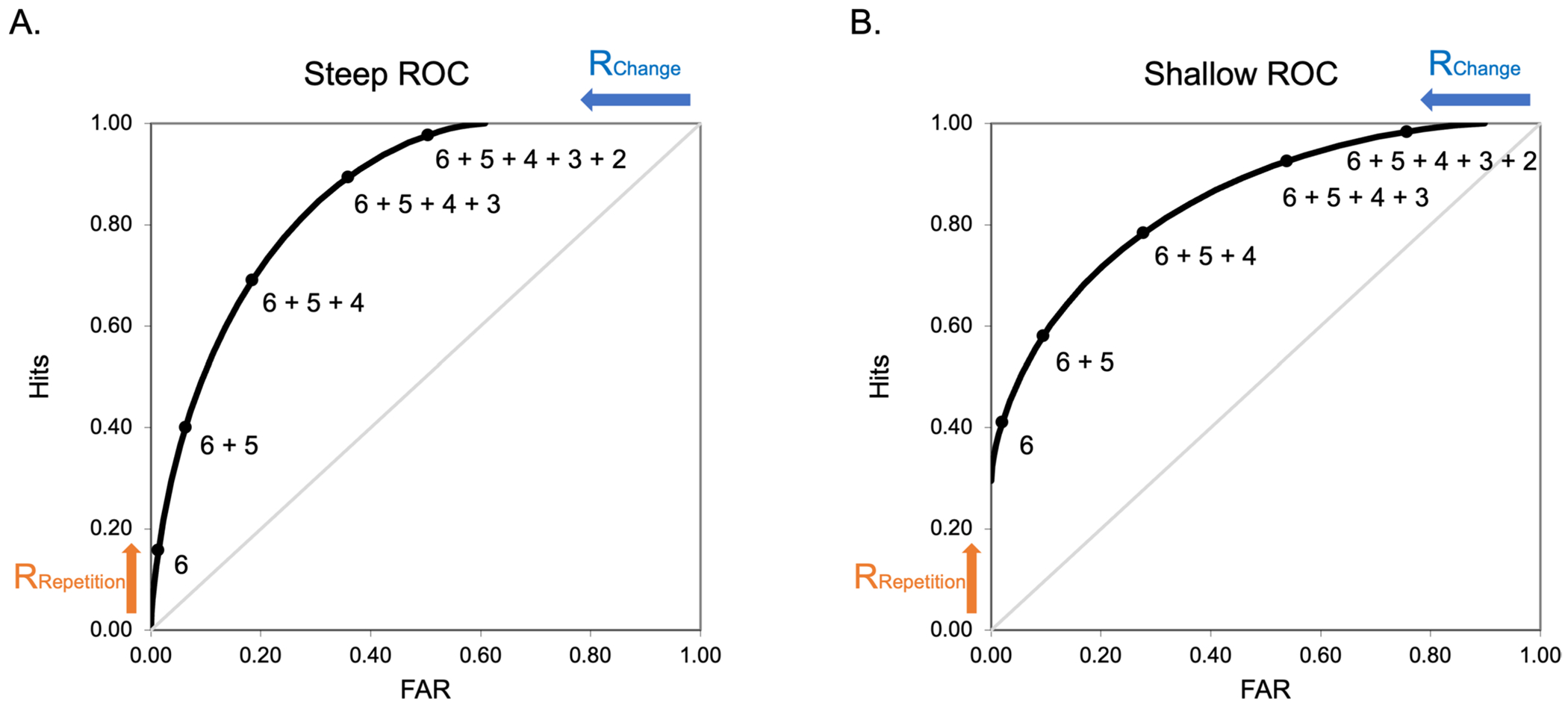
(**A**) An ROC curve with a steep slope, fit to the dual-process model. (**B**) An ROC curve with a shallow slope, fit to the dual-process model

Recognition memory confidence responses can be used to plot ROCs and derive parameter estimates of the underlying memory processes. An ROC relates the proportion of repeated items correctly accepted as the same (i.e., hits) to the proportion of changed items incorrectly accepted as the same (i.e., false alarms) across confidence levels (see Fig. 2A–B). Thus, the left-most point of each ROC reflects the proportion of items receiving a 6 (“sure same”) response; the next point includes 6s and 5s, and so on.

**Fig. 3:**
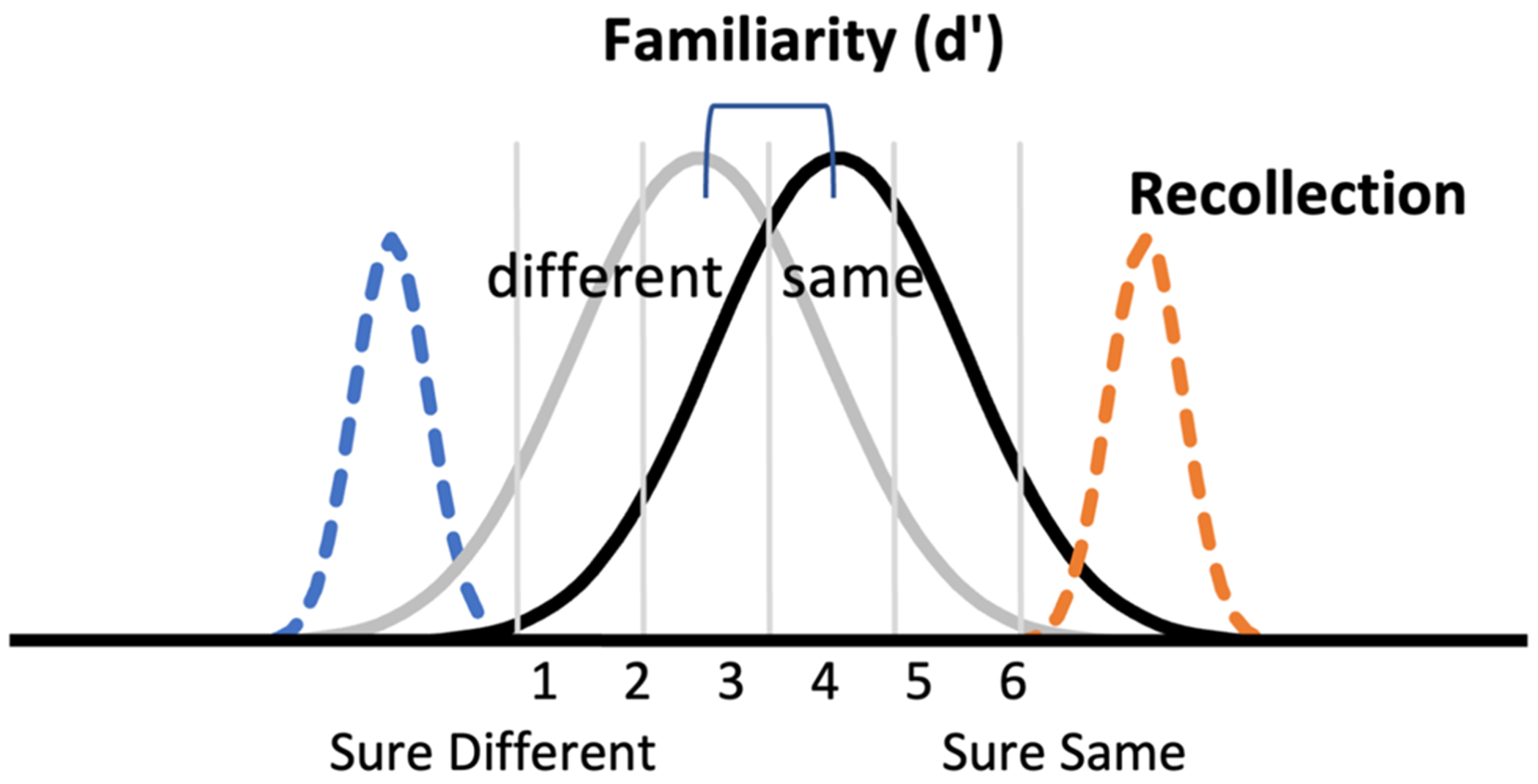
Memory strength distributions in the dual-process model. The gray and black curves show the overlapping familiarity distributions for “same” and “different” items. The dashed blue curve represents recollection-based evidence for detecting changes, producing high-confident “different” responses. The dashed orange curve represents recollection-based evidence for detecting repetitions, producing high-confidence “same” responses

The observed ROC points can be fit to a dual-process model (DPSD) to estimate the contributions of the underlying memory processes. This model (see Fig. 3) assumes that working memory performance reflects a mixture of familiarity- and recollection-based responses (for applications of the model to working memory, see Aly & Yonelinas, 2012; Goodrich et al., 2019; Xie & Zhang, 2017a, 2017b; for applications to episodic memory, see Yonelinas, 1994; Yonelinas & Parks, 2007). The model assumes familiarity reflects the extent to which the test item is perceived as familiar, whereas recollection reflects the ability to actively maintain qualitative information about a study item. It is assumed that repeated items feel more familiar than changed items because repetitions produce a stronger match with the contents of memory. Familiarity is also assumed to vary from item to item, producing two overlapping normal distributions – one for “same” items and one for “different” items – in line with classic signal detection theory (i.e., the gray and black distributions in Fig. 3). This Gaussian memory strength signal is broadly consistent with distributed cortical neural network models that rely on Hebbian learning (e.g., Elfman et al., 2014; Norman & O’Reilly, 2003). Thus, familiarity yields a symmetric, curvilinear ROC whose slope equals 1.0 when plotted in z-space. The distance between the two distributions (*d’*) reflects familiarity strength; as *d’* increases, the ROC becomes more curved and moves farther away from the chance diagonal.

In addition to familiarity, participants can also recollect qualitative details about the study array – for example, actively maintaining that a specific study item was blue. Recollection is treated as a threshold process, meaning that it can sometimes fail (e.g., subjects may have failed to attend to a study item, it was not actively maintained during the retention interval, or there was a retrieval failure). Recollection is thought to depend on frontoparietal attention networks that support the active maintenance of attended items (Yonelinas et al., 2024).

Because recollection carries qualitative information about the attended items, it can impact performance in two ways. First, it can support the detection of changes. That is, if a subject is actively maintaining that an item is blue, and that item then changes to yellow, this should lead to a highly confident “sure different” response. This is illustrated in Fig. 3 as the dashed blue distribution on the left side and will result in an ROC that is not symmetrical but rather will exhibit a steep slope as in Fig. 2A (this ROC would have a slope greater than 1.0 when plotted in z-space). The probability of detecting a change will be estimated by the upper intercept of the resulting ROC, which will move further to the left as change detection increases.

Recollection can also support the detection of repetitions. That is, if a subject is actively maintaining that an item is blue, and that item is then repeated, this should lead to a highly confident “sure same” response. This is illustrated in Fig. 3 as the dashed orange distribution on the right side and will result in an ROC with a shallow slope, as in Fig. 2B (slope of less than 1.0 when plotted in z-space). In this case, recollection of repetitions can be estimated by the left intercept of the ROC, which will move upwards as repetition detection increases.

To derive estimates of each process, the dual-process model is fit to the observed ROC points. This fitting minimizes the sum of squared errors (SSE) between the observed hit and false alarm rates at each confidence level with those predicted by the model. The Excel sheet used to derive parameter estimates is available on the Open Science Framework (OSF; see Data availability in the *Declarations* section), and see also Koen et al. (2017) for a maximum-likelihood estimation toolbox. The DPSD model equations for the hit rate and the false alarm rate are:

$$P\left(\prime \mathrm{Same}\prime |\mathrm{Same}\right)=R_{\mathrm{Repetition}}+\left(1-R_{\mathrm{Repetition}}\right)\times \left(F_{\mathrm{Repetition}}>cr\right)\;\;P\left(\prime \mathrm{Same}\prime |\mathrm{Different}\right)=\left(1-R_{\mathrm{Change}}\right)\times \left(F_{\mathrm{Change}}>cr\right)$$

where R_Repetition_ denotes the probability that the repeated item is recollected, (F_Repetition_ > cr) is the probability that its familiarity signal exceeds a decision criterion, R_Change_ denotes the probability that a changed item is recollected, and (F_Change_ > cr) is the probability that the familiarity of a changed item exceeds the response criterion.

### Experiment 1: Confidence ROCs for *Any-Difference* and *Any-Sameness* Tests

In Experiment 1, we examined visual working memory in *Any-Difference* and *Any-Sameness* tests in which participants rated the confidence of their memory decisions. We then examined ROCs to determine if change detection and repetition detection were differentially engaged in these two tests. We expected change detection to be more useful in the *Any-Difference* test, as detecting a change requires identifying a change in a single item. In contrast, detecting a “same” trial requires maintaining memory for all studied items. Consequently, the resulting ROC should be steep (e.g., similar to Fig. 2A). In contrast, we expected repetition detection to play a larger role in the *Any-Sameness* test. Identifying a repeated item can be accomplished from a single match, whereas determining that a trial is “different” requires maintaining information about the entire study array. Under these conditions, the ROC is expected to be relatively shallow (e.g., similar to Fig. 2B). In addition, parameter estimates were expected to show that the probability of recollecting a change was greater in the *Any-Difference* test than in the *Any-Sameness* test, whereas the probability of recollecting a repetition was greater in the *Any-Sameness* test than in the *Any-Difference* test. Thus, the ROC results in the *Any-Difference* test were expected to replicate the *complex-probe* test results from Norris and Yonelinas (2025). However, as far as we know, ROCs have not been examined in the *Any-Sameness* test, and so the current study provides a novel test of the dual-process model. Finally, based on prior work demonstrating that familiarity contributes to visual working memory (e.g., Aly & Yonelinas, 2012; Norris & Yonelinas, 2025), we expected that familiarity would be useful in discriminating between changed and repeated items, but we did not have a strong prediction about whether it would contribute more to one test than the other. In fact, in Norris and Yonelinas (2025), familiarity contributed equally to all three test types examined.

## Methods

### Participants

Thirty participants were recruited from the undergraduate population at the University of California, Davis, and took part in the study for course credit. All had normal or corrected-to-normal vision, with normal color vision confirmed using the Colormax Ishihara-type color blindness test (https://colormax.org/color-blind-test/). Informed consent was obtained prior to participation. Data collection stopped after reaching 30 participants, with two pre-determined exclusion criteria: (1) each participant had to produce at least three ROC points, and (2) performance had to exceed chance level (i.e., the ROC had to fall above the chance diagonal). Applying these criteria led to the removal of three participants who lacked a sufficient response range to characterize ROC shape (fewer than three ROC points) and another three who performed at chance level. This resulted in a final sample of 24 participants, whose ages ranged from 18 to 23 years.

### Stimuli

The tests were presented on a 17-in. LCD monitor with a resolution of 1,920 × 1,080 pixels. Stimuli were displayed and responses recorded using PsychoPy (v2024.2.2). The colored squares were displayed against a neutral gray background (RGB: 0, 0, 0 in PsychoPy units), with dimensions standardized at 50 × 50 pixels. Table 1 lists the RGB values of the 12 possible color stimuli, along with the corresponding shade changes used for “different” trials in the *Any-Difference* test.

### Procedure

Participants completed the *Any-Difference* and *Any-Sameness* tests on a computer equipped with a standard keyboard and mouse. Confidence responses were collected via keyboard, with number keys mapped to specific response criteria. Testing took place in a single 50 minute session, with thorough instructions given beforehand. Because the two tests were administered in separate blocks, participants first completed five “same” and five “different” practice trials presented in random order before the first test, completed that testing block, and then completed another randomly ordered set of practice trials prior to the second testing block.

For each trial, five colors were randomly selected from the specified “original color RGB value” list (Table 1) to form the study array. On each trial, five candidate square positions were generated by sampling X–Y coordinates from uniform distributions within five predefined screen regions that surrounded, but explicitly excluded, the central fixation area. Each region was associated with exactly one square, ensuring that square centers were always drawn from distinct spatial zones. Because coordinates were independently sampled on each trial, the exact distances between squares varied from trial to trial, introducing spatial jitter across displays. This procedure guaranteed that no square could appear at the fixation location and avoided spatial overlap between squares. The same randomly generated positions were applied to both displays (study and test) within a trial.

Each trial began with a centrally presented fixation cross (+) that remained visible throughout the trial. To counterbalance the two tests, half of the participants completed the *Any-Difference* test first and then the *Any-Sameness* test, while the other half completed them in the reverse order. Analyses of test order revealed no significant impact on performance so order was removed as a factor in the primary analysis. Specifically, for the *Any-Difference* test, performance did not differ between the counterbalanced groups (*t*(19.88) = −1.32, *p* = .20, 95% CI [−0.10, 0.02]), and for the *Any-Sameness* test, the effect of order was likewise nonsignificant (*t*(20.22) = 0.85, *p* = .41, 95% CI [−0.04, 0.10]). Within each test, 80 “same” trials and 80 “different” trials were presented in random order. The study array was presented for 1s, followed by a 500ms fixation screen, and then a non-speeded test array. The test arrays did not cue participants to any particular location; rather, participants had to indicate whether the test array was the “same” as or “different” from the study array. In the *Any-Difference* trials, the sample array was either repeated – with all five colored squares in the same locations (“same” trial) – or one square was randomly changed to a different shade of its original color (“different” trial). In the *Any-Sameness* trials, the test array also preserved square locations and consisted either of five entirely new colors (“different” trial) or four new colors plus one repeated color from the sample array (“same” trial).

Participants made “same/different” judgments using a 6-point confidence scale that was visible at the bottom of the screen throughout the response window (1 = sure different, 2 = probably different, 3 = maybe different, 4 = maybe same, 5 = probably same, 6 = sure same). In the *Any-Difference* test, a “sure different” response indicated that one of the squares had changed its shade, and a “sure same” response indicated that all five squares were the same as in the study array. In the *Any-Sameness* test, a “sure different” response indicated that all five squares were entirely new colors (i.e., the participant detected no repetitions), and a “sure same” response indicated that one square was repeated from the study array. After participants responded, there was a 1s delay before the next trial began.

## Results

### Change and repetition detection ROCs

Performance was measured by examining the observed ROC curves. “Same/different” confidence ratings were used to generate individual ROCs, as well as an aggregate ROC for each condition. The distribution of confidence ratings for each test is provided in Supplementary Table 1 and the raw data are available on OSF (see Data availability in the *Declarations* section).

**Fig. 4:**
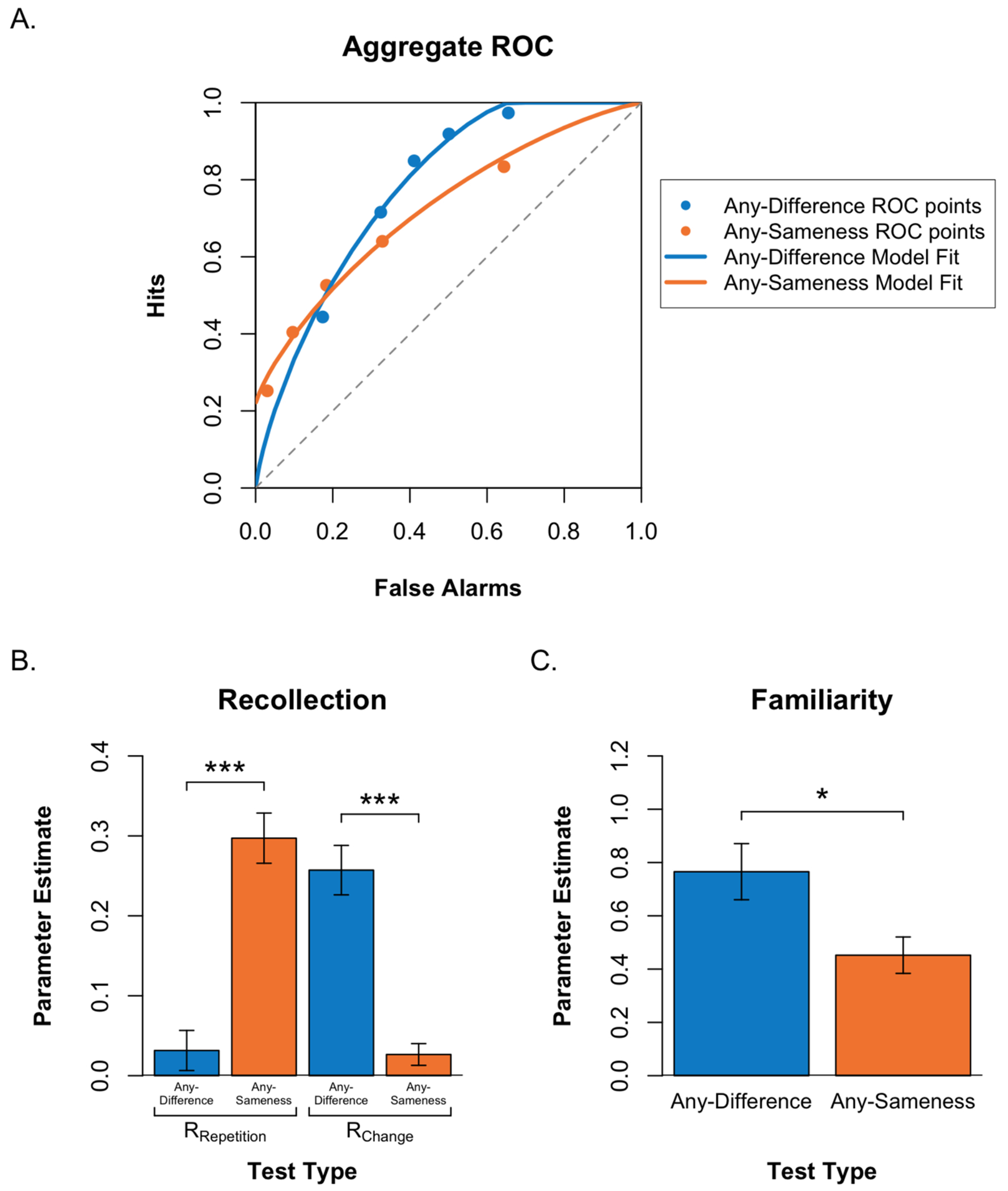
(**A**) Aggregate receiver operating characteristic (ROC) points with model fits. (**B**) Estimates of recollection type. (**C**) Estimates of familiarity. (* *p* < .05, *** *p* < .001)

Consistent with previous VWM ROC studies, both ROCs were curvilinear and exhibited an inverted U-shape (see Fig. 4A). More importantly, as predicted, the *Any-Difference* test exhibited a steep slope, whereas the *Any-Sameness* test exhibited a shallow slope. To quantify this, we plotted the ROCs in *z*-space and estimated the slopes. The *z*-slope was steeper in the *Any-Difference* test (*M* = 1.55, 95% CI [1.36, 1.74]) than in the *Any-Sameness* test (*M* = 0.70, 95% CI [0.61, 0.80]), *F*(1,23) = 61.98, *p* < .001, partial η^2^ = .232. In addition, the slope in the *Any-Difference* test was significantly greater than 1.0, *t*(23) = 5.93, *p* < .001 (*d* = 1.21, 95% CI [0.67, 1.73]), while the slope in the *Any-Sameness* test was significantly less than 1.0, *t*(23) = −6.42, *p* < .001 (*d* = −1.31, 95% CI [−1.85, −0.75]). Overall performance appeared similar across tests, as reflected by the overlapping ROC curves; however, analysis of the area under the curve (AUC) revealed reliably better performance in the *Any-Difference* test (*M* = 0.76, 95% CI [0.73, 0.79]) than in the *Any-Sameness* test (*M* = 0.71, 95% CI [0.67, 0.74]), *F*(1,23) = 15.57, *p* < .001, partial η^2^ = .404, indicating a large effect.

The steep slope in the *Any-Difference* test indicates that participants were more accurate at detecting changes than repetitions (i.e., the right side of the function, which includes more confident “different” responses, is further from the chance diagonal than the left side of the function). In contrast, the shallow slope in the *Any-Sameness* test indicates that participants were more accurate at detecting repetitions than changes (i.e., the left side of the function, which includes the confident “same” responses, is further from the chance diagonal than the right side).

### Parameter Estimates

To further characterize performance, we fit the ROC curves using a dual-process model to estimate the probability of recollecting changes (R_Change_), recollecting repetitions (R_Repetition_), and familiarity-based discrimination (*d’*). The dual-process model provided an excellent fit to the ROC data in both tests (see Fig. 4A). The residual SSE was very low – 0.000986 for the *Any-Difference* test and 0.00120 for the *Any-Sameness* test – showing only minimal discrepancy between observed and predicted hit and false alarm rates in both conditions. A repeated-measures ANOVA on the recollection estimates (Fig. 4B) revealed a strong interaction between test type and recollection type, *F*(1,23) = 66.33, *p* < .001, partial η^2^ = .74, indicating that each test selectively relied on a different form of recollection. Subsequent paired *t*-tests revealed that the interaction was driven by greater recollection of repetitions (R_Repetition_) in the *Any-Sameness* test than in the *Any-Difference* test, *t*(23) = 5.16, *p* < .001, Cohen’s *d* = 1.05, 95% CI [0.5, 2.45], and by greater recollection of changes (R_Change_) in the *Any-Difference* test than in the *Any-Sameness* test, *t*(23) = 9.50, *p* < .001, Cohen’s *d* = 1.94, 95% CI [1.42, 2.99]. Within each test, recollection was also selective: in the *Any-Difference* test, R_Change_ was greater than R_Repetition_, *t*(23) = 7.56, *p* < .001, Cohen’s *d* = 1.54, 95% CI [0.93, 2.85], whereas in the *Any-Sameness* test, R_Repetition_ was greater than R_Change_, *t*(23) = 6.16, *p* < .001, Cohen’s *d* = 1.26, 95% CI [0.77, 2.3].

A repeated-measures ANOVA was conducted to also compare familiarity estimates (*d’*) across tests (see Fig. 4C). The analysis revealed a significant effect of test, *F*(1,23) = 6.97, *p* < .05, partial η^2^ = .23, indicating a large effect, such that familiarity estimates were greater in the *Any-Difference* test (*M* = 0.72, 95% CI [0.54, 0.90]) than in the *Any-Sameness* test (*M* = 0.45, 95% CI [0.31, 0.59]). These results are consistent with the finding that overall discrimination, as measured by AUC, was greater in the *Any-Difference* test than in the *Any-Sameness* test.

In sum, the results of Experiment 1 showed that change detection played a greater role in the *Any-Difference* test, whereas repetition detection played a greater role in the *Any-Sameness* test. Parameter estimates revealed that the enhanced detection of repetitions in the *Any-Sameness* test was driven by an increase in the probability of recollecting the repeated item (R_Repetition_). Conversely, the better detection of changes in the *Any-Difference* test stemmed from an increase in the probability of recollecting that an item had changed (R_Change_).

Importantly, the current results show a clear test-dependent asymmetry: in the *Any-Difference* test, the estimate for R_Repetition_ was near zero, whereas in the *Any-Sameness* test, the estimate for R_Change_ was near zero. This pattern suggests that each test primarily engaged one recollection process over the other. This is consistent with the pattern of results reported by Norris and Yonelinas (2025), where in the *complex-probe* test, R_Repetition_ was quite low, whereas in the *item recognition* test, R_Change_ was also near zero, but the latter reached a value significantly greater than zero (*M* = 0.18, *t*(22) = 3.74, *p* < .001). Nonetheless, it is important to note that in the *single-probe* test, high estimates of both R_Change_ and R_Repetition_ were observed. Taken together, these findings suggest that although test type can bias performance toward one recollection process or the other, both forms of recollection can contribute to VWM decisions (i.e., working memory tests are not entirely process pure).

### Experiment 2: Confidence ROCs and Subjective Reports for *Any-Difference* and *Any-Sameness* Tests

Experiment 2 was designed to determine whether the results of Experiment 1 would replicate in a new sample of participants and whether the same patterns would be observed when recollection and familiarity were estimated using a subjective report procedure. Thus, participants completed both tests, but this time, they provided recognition confidence judgments and subjective reports of “perceiving” and “sensing.”

## Methods

### Participants, stimuli, and procedure

Thirty-seven participants were recruited from the undergraduate population at the University of California, Davis. Four participants were removed due to program error, three more were removed due to a lack of range in responses to evaluate ROC shape (i.e., having less than 3 ROC points), and two were removed because they performed at chance level, giving us a total sample of 28. Participants’ ages ranged from 18 to 23 years. The materials and procedure were identical to those in Experiment 1, with the key addition of subjective report measures. After providing a confidence rating, participants were prompted to choose from one of three options designed to capture their conscious experience of change and repetition detection. For the *Any-Difference* test, the options were: (1) “perceive change,” indicating that they maintained the color of a square and perceived that it had changed to another color; (2) “sense change,” indicating that they felt there was a change without perceiving a specific change; and (3) “no change,” indicating that they perceived all squares as remaining the same. For the *Any-Sameness* test, the corresponding options were: (1) “perceive repetition,” indicating that they maintained the color of a square and perceived that it had not changed; (2) “sense repetition,” indicating that they felt there was a repetition without perceiving a specific item repeat; and (3) “no repetition,” indicating that they perceived all squares as new. Participants were explicitly told that both “perceive” and “sense” responses involved a felt experience of change or repetition, differing only in whether the specific change or repetition could be identified. The distribution of confidence ratings, as well as the “perceiving/sensing” responses for Experiment 2, can be found in Supplementary Tables 2 and 3.

### Analysis

The ROCs were used to derive parameter estimates as described in Experiment 1. In addition, independent of participants’ confidence judgments, reports of “perceiving” and “sensing” were also used to derive parameter estimates (Aly & Yonelinas, 2012; Goodrich et al., 2019). For example, in the *Any-Difference* test, R_Change_ was estimated as P(P_Change_ | Change) – P(P_Change_ | Same). The probability that items were accepted as “different” on the basis of familiarity was estimated as the probability of making a “sense change” response, given that a “perceive change” response was not made, and this was done separately for “same” and “different” trials (i.e., Familiarity_Change_ = P(S_Change_ | Change)/[1-P(P_Change_ | Change)]; Familiarity_Same_ = P(S_Change_ | Same)/[1-P(P_Change_ | Same)]. Familiarity sensitivity (*d’*) was then estimated using the inverse of the standard normal cumulative distribution (i.e., “NORM.S.INV”): *d’* = NORM.S.INV (Familiarity_Change_) – NORM.S.INV(Familiarity_Same_).

In the *Any-Sameness* test, R_Repetition_ was estimated as P(P_Same_ | Same) – P(P_Same_ | Change). The probability that items were accepted as “same” on the basis of familiarity was estimated as the probability of making a “sense same” response, given that a “perceive same” response was not made, and this was done separately for “same” and “different” trials (i.e., Familiarity_Same_ = P(S_Same_ | Same)/[1-P(P_Same_ | Same)]; Familiarity_Change_ = P(S_Same_ | Change)/[1-P(P_Same_ | Change)]. Familiarity sensitivity (*d’*) was then estimated using the inverse of the standard normal cumulative distribution (i.e., “NORM.S.INV”): *d’* = NORM.S.INV(Familiarity_Same_) – NORM.S.INV(Familiarity_Change_).

## Results

**Fig. 5:**
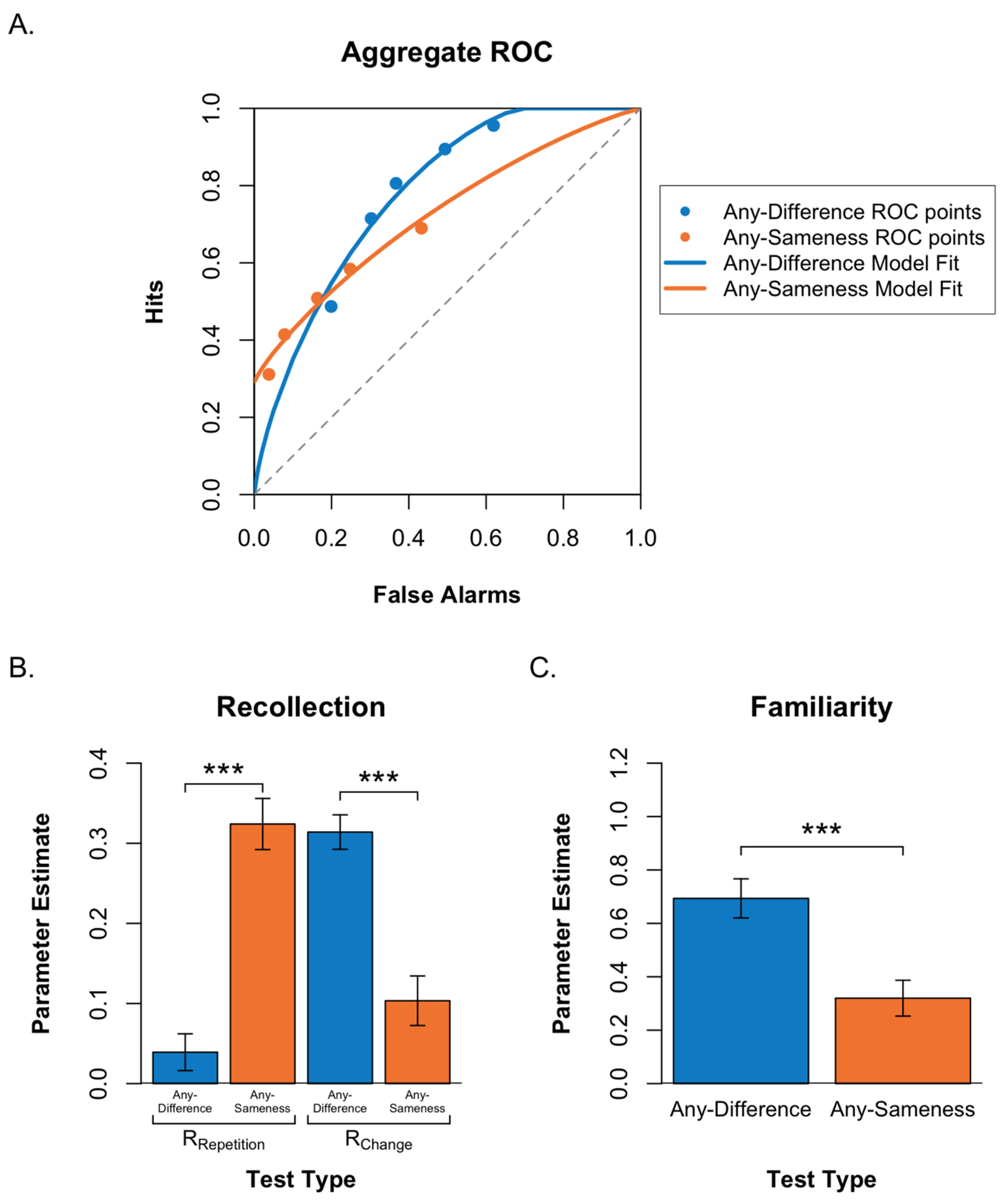
(**A**) Aggregate receiver operating characteristic (ROC) points with model fits (Experiment 2). (**B**) Estimates of recollection type. (**C**) Estimates of familiarity. (*** *p* < .001)

The ROCs closely replicated those reported in Experiment 1, with the *Any-Difference* test producing a steep slope and the *Any-Sameness* test producing a shallow slope (Fig. 5A). To quantify the degree of asymmetry, we plotted the average ROCs in z-space. A repeated-measures ANOVA indicated that there was a significant effect of test type on slope, *F*(1,27) = 180.9, *p* < .001, partial η^2^ = .51. In the *Any-Difference* test, the mean z-slope was 1.57 (95% CI [1.44, 1.71]), which was significantly greater than 1.0, *t*(27) = 8.63, *p* < .001 (*d* = 1.63, 95% CI [1.05, 2.19]). Whereas, in the *Any-Sameness* test, the mean z-slope was 0.56 (95% CI [0.49, 0.63]), which was significantly less than 1.0, *t*(27) = −13.76, *p* < .001 (*d* = −2.60, 95% CI [−3.38, −1.81]). Although overall performance appeared similar across tests, a repeated-measures ANOVA revealed a significant effect of test on AUC, *F*(1,27) = 21.6, *p* < .001, partial η^2^ = .44, indicating a large effect. Specifically, performance was higher in the *Any-Difference* test (*M* = 0.76, 95% CI [0.73, 0.79]) than in the *Any-Sameness* test (*M* = 0.71, 95% CI [0.68, 0.73]).

### Parameter Estimates

Similarly to Experiment 1, the dual-process model provided an excellent fit to the ROC data in both tests (see Fig. 5A). The residual SSE was very low – 0.00169 for the *Any-Difference* test and 0.00124 for the *Any-Sameness* test – showing only minimal discrepancy between observed and predicted hit and false alarm rates in both conditions. Examining the parameter estimates for recollection (see Fig. 5B), a repeated-measures ANOVA revealed a significant interaction between test type and type of recollection (*F*(1,27) = 88.87,*p* < .001), partial η^2^ = .77, replicating that each test selectively relied on a different form of recollection. Subsequent *t*-tests confirmed that this interaction arose because recollecting a repetition (R_Repetition_) occurred more often in the *Any-Sameness* test, *t*(27) = 9.38, *p* < .001, Cohen’s *d* = 1.77, 95% CI [1.13, 2.91], whereas recollecting a change (R_Change_) occurred more often in the *Any-Difference* test, *t*(27) = 5.73, *p* < .001), Cohen’s *d* = 1.08, 95% CI [0.67, 1.74]. Additionally, in the *Any-Difference* test, R_Change_ was significantly higher than R_Repetition_ (*t*(27) = 8.31, *p* < .001), Cohen’s *d* = 1.57, 95% CI [1.18, 2.3], and in the *Any-Sameness* test, R_Repetition_ was greater than R_Change_ (*t*(27) = 5.97, *p* < .001), Cohen’s *d* = 1.13, 95% CI [0.66, 2.06]. A repeated-measures ANOVA compared familiarity estimates (*d’*) across the two tests (see Fig. 5C). The analysis revealed a significant effect, *F*(1,27) = 27.57, *p* < .001, partial η^2^ = .505, such that familiarity estimates were significantly higher in the *Any-Difference* test (*M* = 0.69, 95% CI [0.54, 0.84]) compared to the *Any-Sameness* test (*M* = 0.32, 95% CI [0.18, 0.45]).

Thus, as in Experiment 1, the ROC parameter estimates indicated that the enhanced ability to detect repetitions in the *Any-Sameness* test reflected an increase in the probability that participants actively maintained the repeated items (i.e., R_Repetition_), whereas the enhanced ability to detect changes in the *Any-Difference* test reflected an increase in the probability that participants actively maintained the changed items (i.e., R_Change_).

### Subjective Reports

**Fig. 6:**
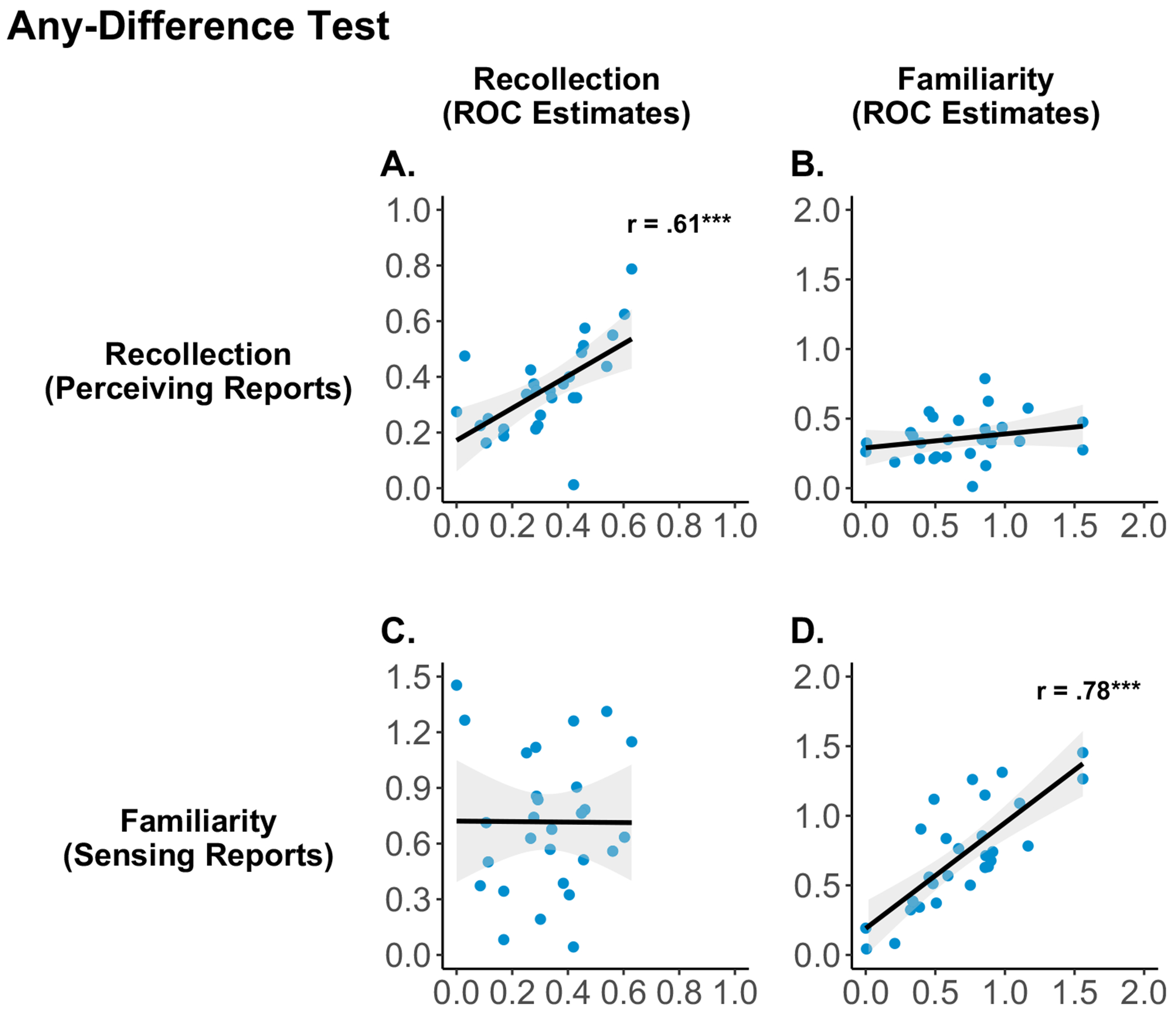
Correlations between receiver operating characteristic (ROC) estimates and subjective report estimates in the *Any-Difference* test. (**A, B**) Recollection and familiarity plotted against estimates of recollection based on “perceive change” reports. (**C, D**) Recollection and familiarity plotted against familiarity estimates based on “sense change” reports. Shaded areas represent 95% confidence intervals

**Fig. 7:**
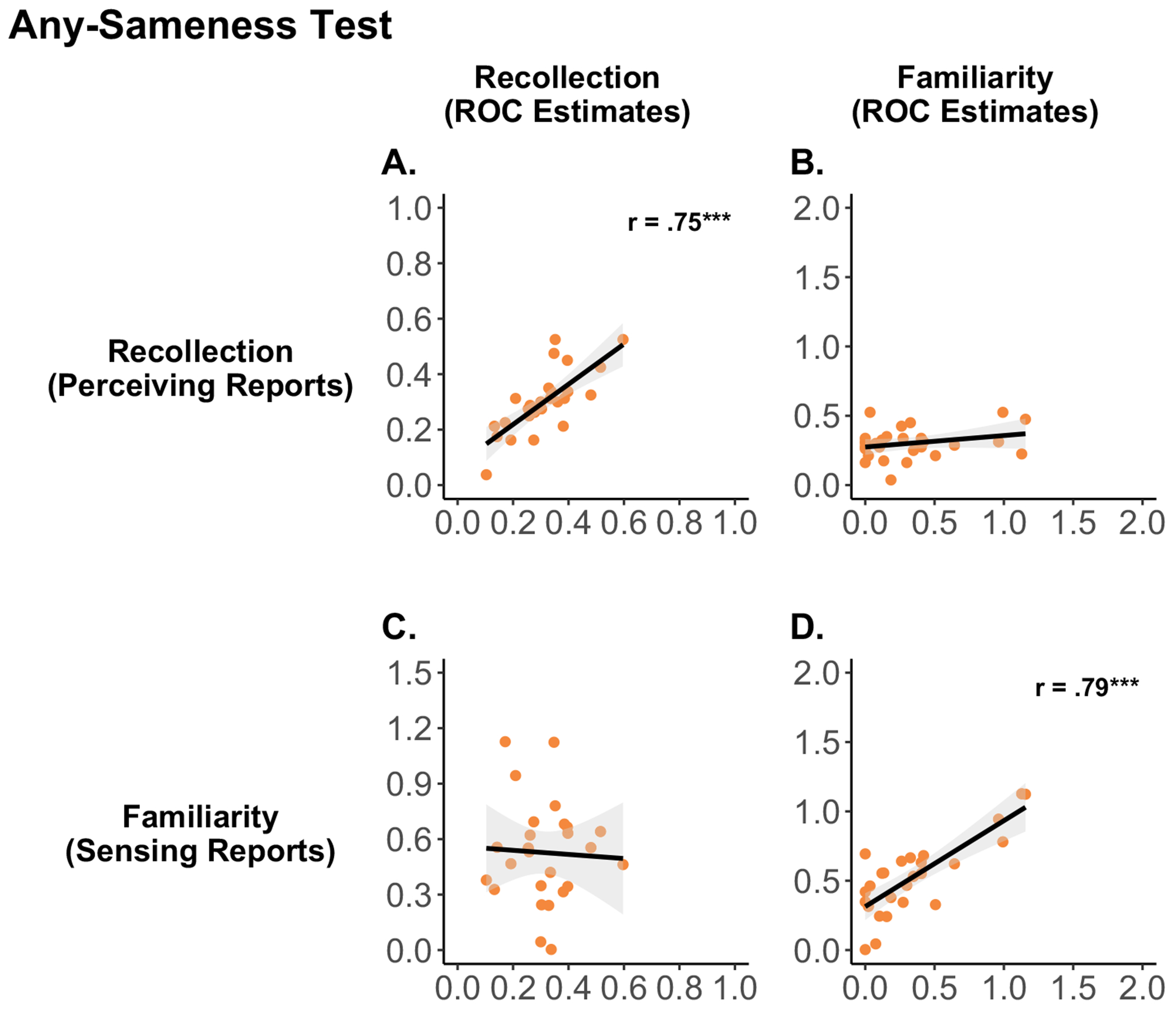
Correlations between receiver operating characteristic (ROC) estimates and subjective report estimates in the *Any-Sameness* test. (**A, B**) Recollection and familiarity plotted against estimates of recollection based on “perceive repetition” reports. (**C, D**) Recollection and familiarity plotted against familiarity estimates based on “sense repetition” reports. Shaded areas represent 95% confidence intervals

To quantify the relationship between subjective reports and ROC estimates, we examined correlations between these measures. For the *Any-Difference* test, Pearson correlations revealed significant positive relationships between recollection based on subjective reports of “perceive change” and ROC estimates of R_Change_, *r*(26) = .61, 95% CI [0.30, 0.80], *t* = 3.89, *p* < .001 (see Fig. 6A), but not with ROC estimates of familiarity, *r*(26) = .24, 95% CI [−0.15, 0.56], *t* = 1.25, *p* > .05 (Fig. 6B). Conversely, familiarity based on subjective reports of “sense change” were not correlated with ROC estimates of R_Change,_ *r*(26) = −.01, 95% CI [−0.38, 0.37], *t* = −0.03, *p* > .05 (Fig. 6C), but they were significantly correlated with ROC estimates of familiarity, *r*(26) = .78, 95% CI [0.57, 0.89], *t* = 6.30, *p* < .001 (Fig. 6D). Similarly, for the *Any-Sameness* test, significant positive relationships were found between recollection based on subjective reports of “perceive repetition” and ROC estimates of R_Repetition_, *r*(26) = .75, 95% CI [0.52, 0.88], *t* = 5.79, *p* < .001 (see Fig. 7A), but not with ROC estimates of familiarity, *r*(26) = .27, 95% CI [−0.12, 0.58], *t* = 1.40, *p* > .05 (Fig. 7B). Conversely, familiarity based on subjective reports of “sense repetition” were not correlated with ROC estimates of R_Repetition_, *r*(26) = −.05, 95% CI [−0.42, 0.33], *t* = −0.26, *p* >.05 (Fig. 7C), but they were significantly correlated with ROC estimates of familiarity, *r*(26) = .79, 95% CI [0.58, 0.90], *t* = 6.49, *p* < .001 (see Fig. 7D).

The finding that ROC estimates of recollection were directly related to reports of “perceiving” and not reports of “sensing,” and that the ROC estimates of familiarity were related to reports of “sensing” rather than “perceiving,” indicates that the ROC modeling was effectively measuring recollection and familiarity. Overall, the findings further support the conclusion that the ability to detect changes and repetitions reflect an increase in the ability to actively maintain items in working memory (i.e., recollection).

## Discussion

The current set of experiments examined the roles of change detection and repetition detection in visual working memory utilizing *Any-Difference* and *Any-Sameness* tests. Although prior work has suggested that these two forms of recollection can be functionally dissociated across common working memory tests (Norris & Yonelinas, 2025), the current study is the first to test for this dissociation by directly manipulating whether the test requires change detection or repetition detection. In addition, the current studies measured the roles of recollection and familiarity using both confidence ROC modeling and subjective reports of “perceiving” and “sensing” to verify that the observed increases in accurate change detection and repetition detection were related to the subjects’ ability to recollect mnemonic information across the delay interval, rather than simply reflecting differences in familiarity.

Experiment 1 used confidence responses to examine the roles of change detection and repetition detection. As expected, the *Any-Difference* test led to disproportionately more accurate high-confidence “different” responses for changed trials and resulted in a steep ROC slope (z-slope > 1), indicating that the test relied more heavily on change detection. In contrast, the *Any-Sameness* test led to disproportionately more accurate high-confidence “same” responses for repeated trials and resulted in a shallow slope (z-slope < 1), indicating that the test relied moreso on repetition detection. To further quantify these effects, the ROCs were fit with a dual-process model, which yielded parameter estimates showing that recollection of changes occurred primarily in the *Any-Difference* test, whereas recollection of repetitions occurred primarily in the *Any-Sameness* test.

Experiment 2 was similar to Experiment 1, except that subjects were required to provide not only confidence judgments but also subjective reports of “perceiving” and “sensing.” The confidence results closely replicated those of Experiment 1. In addition, subjective reports converged with ROC-based parameter estimates: recollection estimates were significantly correlated with estimates based on “perceiving” but not “sensing,” whereas familiarity estimates were significantly correlated with estimates based on “sensing” but not “perceiving.” These results indicate that recollection and familiarity do not merely reflect differences in memory strength or confidence but instead are processes accessible to awareness.

Notably, this convergence between subjective reports and ROC estimates replicates prior findings with complex materials such as scenes (Aly & Yonelinas, 2012) and faces (Goodrich et al., 2019) and extends them to minimalistic colored-square arrays. Thus, subjective reports provide a useful method for assessing recollection and familiarity in working memory, even with simple stimuli commonly used in laboratory studies.

The current results replicate and extend the work of Norris and Yonelinas (2025), which showed that recollection of changes played a particularly dominant role in *complexprobe* tests, whereas the recollection of repetitions played a dominant role in *item recognition* tests. However, these earlier tests differ in the degree to which they require location and color information, as well as whether they use single items or complex arrays as retrieval cues. The current tests differ with respect to the demand for change detection and repetition detection but hold constant the test demands and the number of retrieval cues. As such, the current results provide further evidence that recollection supports both change detection and repetition detection in functionally dissociable ways.

One potentially interesting difference across these studies was that in the original study (Norris & Yonelinas, 2025), familiarity remained constant across tests, whereas in the current study, familiarity estimates were higher in the *Any-Difference* test than in the *Any-Sameness* test. This could be due to the fact that in the *Any-Difference* test, subjects were more willing to make use of familiarity information than in the *Any-Sameness* test (e.g., a single change may lead to a more salient change in familiarity than a single repetition). However, it could also be due to differences in overall test difficulty. That is, in the current experiments, overall performance was greater in the *Any-Difference* than the *Any-Sameness* test, which could have led to greater familiarity estimates. In contrast, in Norris and Yonelinas (2025), overall test difficulty was comparable and familiarity contributed similarly to each test. Thus, in general, familiarity does not differentially support change and repetition detection, but future studies will be needed to determine if there are conditions in which it may begin to play a more pronounced role.

Another question that should be addressed in future studies is the extent to which familiarity, change detection, and repetition detection contribute to working memory tests that differentially require item information versus binding information. That is, in the current tests, recognition responses could be based either on item information (i.e., recollecting color) or on binding information (i.e., recollecting that a color was in a specific location). The extent to which these processes support memory for item and binding information is not yet clear.

A reviewer (Nelson Cowan) highlighted a noteworthy parallel with an earlier working memory study by Cowan et al. (2016) in which subjects indicated the number of colors that changed between the study and test arrays (0–5 changes). Although that study did not assess confidence and so did not estimate recollection or familiarity, performance patterns suggested that the proportion of trials in which subjects correctly indicated no changes (− 0.1) was similar to our estimate of repetition detection (− 0.07), whereas the proportion of trials correctly identified as having at least one change (− 0.45) was similar to our estimate of change detection (− 0.43). Although there were many differences between the two paradigms, the numerical similarities suggest that both paradigms tap a comparable underlying memory signal. Accordingly, we interpret this convergence as tentative yet supportive evidence for the robustness of the recollection effects across different test implementations.

### Practical Implications

The current results have important practical implications. They show that change detection and repetition detection are functionally distinct, with their contributions depending critically on the mnemonic demands of the specific working memory test. This suggests that in addition to familiarity, working memory relies on two separate forms of recollection. Future research should explore how other factors – such as stimulus complexity, set size, encoding, or delay – affect these recollection and familiarity processes. In addition, the *Any-Difference* and *Any-Sameness* tests examined here should be useful in future studies that aim to further characterize the role of change detection and repetition detection. For example, this distinction may clarify working memory differences in populations such as older adults or individuals with neurological conditions, who might exhibit selective deficits in either change detection or repetition detection, thereby improving diagnostic accuracy and personalized intervention. Additionally, these behavioral dissociations raise the question of whether these processes are supported by distinct neural networks. Although neuroimaging studies have yet been used to examine these two processes in working memory, an episodic memory study (Bowman & Dennis, 2016), suggested that frontoparietal regions were more active when subjects engaged in recollection rejection (i.e., change detection) than when they engaged in target recollection (i.e., repetition detection). Investigating whether repetition detection and change detection in working memory also reflect partially distinct neural processes will be an important next step.

### Other memory theories

The current results were fit well by a dual-process model in which working memory reflects a mixture of recollection- and familiarity-based responses. The familiarity process is assumed to reflect a Gaussian strength signal that is consistent with signal detection theory (e.g., Wickelgren, 1968) and with a variety of activation and distributed cortical neural network models of working memory (Compte et al., 2000; Elfman et al., 2014; Fuster & Alexander, 1971; Goldman-Rakic, 1992; Norman & O’Reilly, 2003). In contrast, recollection is conceptualized as an active maintenance process that is thresholded and so can sometimes fail. It is assumed to rely on frontoparietal attention networks that maintain the active representation of attended items and is consistent with capacity-limited models of working memory (e.g., Baddeley, 1986; Logie, 1995; Luck & Vogel, 1997).

The finding that both recollection and familiarity contribute to working memory argues against single-component working memory models – such as single-parameter threshold models often used to estimate capacity and standard signal detection models often used to measure sensitivity (*d’*) – because without additional assumptions, these models cannot account for the observed ROC results and dissociations. For example, threshold models yield only linear ROCs, whereas standard signal detection models yield only symmetrical, curved ROCs (see Kellen & Klauer, 2015; Rouder et al., 2011; Yonelinas & Parks, 2007). One alternative two-component approach that is broadly consistent with the current ROC results is the unequal-variance signal detection (UVSD) model (Hautus et al., 2021; Yonelinas & Parks, 2007), which has a familiarity parameter and an additional variance parameter that can increase the variance of the target or lure familiarity distributions. It can produce ROCs that are similar to those of the dual-process model and thus can produce steep and shallow ROCs (Yonelinas & Parks, 2007). In the UVSD model, differences in variance between “same” and “different” distributions could reflect a recollection-like process, leading to conclusions that would be similar to those from the dual-process model. Other interpretations of this extra-variance component – such as encoding variability (Wixted, 2007) or mechanisms proposed by global matching models (Gillund & Shiffrin, 1984; Hintzman, 1988; Shiffrin & Steyvers, 1997) – may also be plausible but have not been assessed in the context of working memory ROCs.

The dual-process model shares similarities with several other multiple-component working memory theories, but it is not yet clear whether they can produce the types of ROCs, or the subjective reports, that we observed in the current studies. For example, Cowan’s focus-of-attention model (Cowan et al., 2021) and Oberauer’s direct access/context-frame model (Lin & Oberauer, 2022; Oberauer, 2002, 2005, 2013; Oberauer & Lin, 2017) both include an active maintenance process that could support high-confidence working memory judgments (i.e., recollection) and an activation process that could support a signal detection-like familiarity signal. Although, as far as we know, neither approach explicitly addresses the functional separability of change versus repetition detection, nor have they been applied directly to ROC or subjective report results. Other approaches, such as guessing/noise models (Bays et al., 2009; Luck & Vogel, 2013; Schurgin et al., 2020) also include different types of memory signals, but it is unclear how they would accommodate the current results.

Our results converge with a long history of long-term episodic memory studies that have indicated distinct roles for recollection- and familiarity-based responses (Atkinson & Juola, 1974; Jacoby, 1991; Jacoby & Dallas, 1981; Mandler & Rabinowitz, 1981). This convergence may not be too surprising given that the working memory and episodic memory tests differ primarily in terms of the delay periods examined. Although theories of working memory and episodic memory are often quite different, the current results point to a bridge between the literatures and indicate that similar recollection and familiarity processes may contribute to both forms of memory.

## Conclusion

To examine the distinct contributions of change detection and repetition detection to working memory, we compared performance on two visual working memory tests that were designed to selectively engage each process (*Any-Difference* and *Any-Sameness* tests). Analyses of confidence ROCs indicated that accurate performance in the *Any-Difference* test was driven primarily by the recollection of changes, whereas accurate performance in the *Any-Sameness* test was driven primarily by the recollection of repetitions. In addition, analyses of subjective reports of “perceiving” and “sensing” further showed that participants were aware of these processes and could reliably report their occurrence.

## Supplementary Material

26MemorysDoubleTakeSupp

The online version contains supplementary material available at https://doi.org/10.3758/s13421-026-01890-6.

## Figures and Tables

**Table 1:** PsychoPy RGB [−1:1] values of color stimuli

Color	Original RGB values			Shade change RGB values		
	R	G	B	R	G	B
Green	−1.0	1.0	−0.6	−1.0	0.4	−0.6
Yellow	1.0	1.0	−1.0	1.0	0.4	−1.0
Red	1.0	−0.6	−1.0	0.4	−0.6	−1.0
Pink	1.0	−1.0	0.2	1.0	−1.0	0.8
Purple	0.2	−1.0	1.0	0.2	−1.0	0.4
Blue	−1.0	−0.6	1.0	−1.0	−0.6	0.4
Cyan	−1.0	1.0	1.0	−1.0	1.0	0.4
Brown	0.1765	−0.4118	−1.0	0.1765	−0.4118	−0.4
Orange	1.0	0.0980	−1.0	1.0	0.0980	−0.4
Beige	0.6912	0.6931	0.5	0.6912	0.6931	−0.1
Dark green	−1.0	−0.2157	−1.0	−0.4	−0.2157	−1.0
Light pink	1.0	0.5	0.6	1.0	0.5	0

To ensure consistency across colors, either the R, G, or B value was changed by the same amount (either + or − 0.6) to make up the “shade change RGB values.” The RGB values listed are intended as approximations of the stimulus colors. The exact hue that participants perceived could vary with monitor calibration, ambient lighting, and the viewer’s head position and viewing angle during the experiment

## Data Availability

The datasets generated during and/or analyzed during the current study are available on the Open Science Framework (OSF) at: https://osf.io/v6245/overview?view_only=11f5c31cedca496499d9aa5ab724e370.

## Footnotes

1 We thank Dr. Steve Luck for alerting us to this study.

## Code availability

The code to run the analyses is not available.
