# Supplementary material for: Memory’s double take: Dissociating two forms of recollection in visual working memory
Source: Mem Cognit. Author manuscript; Available in PMC 2026 Jul 16. (PMC13375162; doi:10.3758/s13421-026-01890-6)
Supplement: 26MemorysDoubleTakeSupp [file NIHMS2190944-supplement-26MemorysDoubleTakeSupp.docx]

**Supplementary Materials**

**Supplementary Table 1.** Number of responses at each level of confidence for Experiment 1 (N = 24).

| Trial Type | 1 | 2 | 3 | 4 | 5 | 6 |
| --- | --- | --- | --- | --- | --- | --- |
| Difference Present | 662 | 296 | 173 | 166 | 289 | 334 |
| Difference Absent | 51 | 105 | 134 | 256 | 522 | 852 |
| Sameness Absent | 684 | 605 | 278 | 168 | 127 | 58 |
| Sameness Present | 319 | 372 | 219 | 234 | 292 | 484 |

**Supplementary Table 2.** Number of responses at each level of confidence for Experiment 2 (N = 28).

| Trial Type | 1 | 2 | 3 | 4 | 5 | 6 |
| --- | --- | --- | --- | --- | --- | --- |
| Difference Present | 853 | 281 | 284 | 144 | 232 | 446 |
| Difference Absent | 99 | 137 | 199 | 204 | 510 | 1091 |
| Sameness Absent | 1271 | 413 | 190 | 190 | 91 | 85 |
| Sameness Present | 695 | 237 | 169 | 211 | 231 | 697 |

**Supplementary Table 3.** Number of subjective responses per test type for Experiment 2 (N = 28).

| Trial Type | Perceive Change/Repetition | Sense Change/Repetition | No Change/Repetition |
| --- | --- | --- | --- |
| Difference Present | 908 | 591 | 741 |
| Difference Absent | 103 | 431 | 1706 |
| Sameness Absent | 120 | 395 | 1725 |
| Sameness Present | 875 | 480 | 965 |
